# Supplementary material for: Association of plasma acylcarnitines and amino acids with hypertension: A nationwide metabolomics study
Source: PLoS One. 2023 Jan 17;18(1):e0279835. doi: 10.1371/journal.pone.0279835 (PMC9844860; doi:10.1371/journal.pone.0279835)
Supplement: S1 Table — (DOCX) [file pone.0279835.s001.docx]

S1 Table. The multicollinearity test between total cholesterol, triglyceride, and HDL cholesterol according to Pearson correlation coefficient.

| **Variables** | | **Total cholesterol** | **Triglyceride** | **HDL cholesterol** |
| --- | --- | --- | --- | --- |
| **Total cholesterol** | Pearson correlation | 1 | 0.369 | 0.194 |
|  | p-value | - | <0.001 | <0.001 |
| **Triglyceride** | Pearson correlation | .369 | 1 | -0.395 |
|  | p-value | <0.001 | - | <0.001 |
| **HDL cholesterol** | Pearson correlation | 0.194 | -0.395 | 1 |
|  | p-value | <0.001 | <0.001 | - |
