# Supplementary material for: Association of plasma acylcarnitines and amino acids with hypertension: A nationwide metabolomics study
Source: PLoS One. 2023 Jan 17;18(1):e0279835. doi: 10.1371/journal.pone.0279835 (PMC9844860; doi:10.1371/journal.pone.0279835)
Supplement: S2 Table — (DOCX) [file pone.0279835.s002.docx]

S2 Table. The pairwise comparison of sociodemographic and laboratory parameters between groups.

| **Variables** | **Normal vs Elevated BP** | **Normal vs Stage 1 HTN** | **Normal vs Stage 2 HTN** | **Elevated BP vs Stage 1 HTN** | **Elevated BP vs Stage 2 HTN** | **Stage 1 HTN vs Stage 2 HTN** |
| --- | --- | --- | --- | --- | --- | --- |
| **Age (year)** | 0.003 | 0.339 | <0.001 | 0.243 | 0.001 | <0.001 |
| **BMI (kg/m2)** | 0.325 | 0.026 | <0.001 | 1.000 | 0.414 | 0.250 |
| **WC (cm)** | 0.113 | 0.012 | <0.001 | 1.000 | 0.022 | 0.001 |
| **HC (cm)** | 0.673 | 0.218 | <0.001 | 1.000 | 0.607 | 0.183 |
| **SBP (mm Hg)** | <0.001 | <0.001 | <0.001 | 0.018 | <0.001 | <0.001 |
| **DBP (mm Hg)** | <0.001 | <0.001 | <0.001 | <0.001 | <0.001 | <0.001 |
| **FPG (mg/dL)** | 0.734 | 0.005 | <0.001 | 1.000 | 0.257 | 1.000 |
| **HbA1C (%)** | 1.000 | 0.011 | <0.001 | 1.000 | 0.157 | 0.909 |
| **TG (mg/dL)** | 0.479 | 0.012 | 0.001 | 1.000 | 1.000 | 1.000 |
| **Cholesterol (mg/dL)** | 0.327 | <0.001 | <0.001 | 0.862 | 0.464 | 1.000 |
| **Non-HDL-C (mg/dL)** | 0.723 | <0.001 | <0.001 | 0.145 | 0.168 | 1.000 |
| **Smoking, n (%)** | 0.482 | 0.021 | <0.001 | 1.000 | 1.000 | 1.000 |
| **Medications** |  |  |  |  |  |  |
| **Antihypertensive drugs, n (%)** | 0.139 | 1.000 | <0.001 | 0.622 | <0.001 | <0.001 |
| **Oral glucose-lowering drugs, n (%)** | 0.195 | 1.000 | 0.001 | 0.664 | 1.000 | 0.010 |
| **Statins, n (%)** | 0.583 | 1.000 | 0.024 | 1.000 | 1.000 | 0.345 |
