# Supplementary material for: Association of plasma acylcarnitines and amino acids with hypertension: A nationwide metabolomics study
Source: PLoS One. 2023 Jan 17;18(1):e0279835. doi: 10.1371/journal.pone.0279835 (PMC9844860; doi:10.1371/journal.pone.0279835)
Supplement: S3 Table — (DOCX) [file pone.0279835.s003.docx]

S3 Table. The logistic regression analysis on the metabolite profile for predicting Elevated BP, stage 1 HTN, and stage 2 HTN

| parameters | Groups | Crude | | Model 1* | | Model 2** | |
| --- | --- | --- | --- | --- | --- | --- | --- |
|  |  | **OR (95%CI)** | **P value** | **OR (95%CI)** | **P value** | **OR (95%CI)** | **P value** |
| C0 | Elevated BP | 0.85 (0.70- 1.041) | 0.120 | 0.75 (0.61- 0.93) | **0.008** | 0.71 (0.57- 0.88) | **0.003** |
|  | Stage 1 HTN | 1.00 (0.85- 1.17) | 0.975 | 0.91 (0.77- 1.08) | 0.314 | 0.84 (0.70- 1.006) | 0.058 |
|  | Stage 2 HTN | 1.04 (0.89- 1.21) | 0.585 | 0.84 (0.71- 0.99) | **0.040** | 0.75 (0.63- 0.90) | **0.003** |
| C2 | Elevated BP | 0.89 (0.72- 1.10) | 0.300 | 0.80 (0.65- 1.00) | 0.052 | 0.80 (0.64- 0.99) | **0.043** |
|  | Stage 1 HTN | 1.07 (0.91- 1.25) | 0.386 | 1.02 (0.86- 1.20) | 0.795 | 0.98 (0.83- 1.16) | 0.876 |
|  | Stage 2 HTN | 1.20 (1.04- 1.40) | **0.012** | 1.00 (0.85- 1.17) | 0.962 | 0.95 (0.80- 1.13) | 0.612 |
| C3 | Elevated BP | 0.97 (0.79- 1.19) | 0.784 | 0.87 (0.70- 1.08) | 0.208 | 0.83 (0.66- 1.04) | 0.114 |
|  | Stage 1 HTN | 1.22 (1.04- 1.43) | **0.013** | 1.12 (0.95- 1.32) | 0.171 | 1.06 (0.89- 1.27) | 0.477 |
|  | Stage 2 HTN | 1.30 (1.12- 1.51) | **0.001** | 1.07 (0.91- 1.26) | 0.385 | 1.01 (0.84- 1.21) | 0.880 |
| C3DC | Elevated BP | 0.86 (0.70- 1.06) | 0.182 | 0.82 (0.65- 1.01) | 0.074 | 0.80 (0.64- 1.00) | 0.050 |
|  | Stage 1 HTN | 1.16 (0.99- 1.37) | 0.056 | 1.13 (0.96- 1.34) | 0.126 | 1.09 (0.92- 1.30) | 0.288 |
|  | Stage 2 HTN | 1.22 (1.05- 1.42) | **0.008** | 1.11 (0.94- 1.30) | 0.198 | 1.03 (0.87- 1.23) | 0.680 |
| C4 | Elevated BP | 0.92 (0.74- 1.15) | 0.501 | 0.86 (0.69- 1.07) | 0.201 | 0.85 (0.68- 1.06) | 0.152 |
|  | Stage 1 HTN | 1.05 (0.89- 1.23) | 0.540 | 0.99 (0.85- 1.16) | 0.969 | 0.96 (0.82- 1.12) | 0.603 |
|  | Stage 2 HTN | 1.08 (0.93- 1.26) | 0.262 | 0.94 (0.81- 1.11) | 0.513 | 0.89 (0.76- 1.06) | 0.205 |
| C4OH | Elevated BP | 0.96 (0.78- 1.19) | 0.749 | 0.88 (0.71- 1.10) | 0.278 | 0.88 (0.71- 1.10) | 0.275 |
|  | Stage 1 HTN | 1.10 (0.93- 1.29) | 0.243 | 1.05 (0.89- 1.23) | 0.545 | 1.05 (0.88- 1.24) | 0.570 |
|  | Stage 2 HTN | 1.26 (1.08- 1.46) | **0.003** | 1.06 (0.91- 1.25) | 0.418 | 1.05 (0.89- 1.25) | 0.534 |
| C4DC | Elevated BP | 1.15 (0.93- 1.42) | 0.184 | 1.06 (0.86- 1.33) | 0.546 | 1.04 (0.81- 1.33) | 0.751 |
|  | Stage 1 HTN | 1.20 (1.02- 1.42) | **0.026** | 1.14 (0.96- 1.35) | 0.120 | 1.02 (0.84- 1.24) | 0.808 |
|  | Stage 2 HTN | 1.43 (1.23- 1.67) | **<0.001** | 1.21 (1.03- 1.43) | **0.021** | 1.10 (0.90- 1.34) | 0.327 |
| C5 | Elevated BP | 0.91 (0.74- 1.12) | 0.408 | 0.85 (0.68- 1.06) | 0.153 | 0.81 (0.64- 1.01) | 0.069 |
|  | Stage 1 HTN | 1.10 (0.94- 1.29) | 0.227 | 1.01 (0.86- 1.20) | 0.850 | 0.94 (0.79- 1.12) | 0.531 |
|  | Stage 2 HTN | 1.21 (1.04- 1.41) | 0.**010** | 1.10 (0.93- 1.29) | 0.260 | 0.98 (0.82- 1.17) | 0.870 |
| C5:1 | Elevated BP | 1.00 (0.81- 1.23) | 0.982 | 0.96 (0.77- 1.18) | 0.707 | 0.91 (0.73- 1.13) | 0.412 |
|  | Stage 1 HTN | 1.11 (0.94- 1.30) | 0.191 | 1.09 (0.92- 1.28) | 0.295 | 1.02 (0.86- 1.21) | 0.784 |
|  | Stage 2 HTN | 1.14 (0.98- 1.33) | 0.072 | 1.06 (0.91- 1.25) | 0.420 | 0.97 (0.82- 1.16) | 0.799 |
| C5OH | Elevated BP | 0.95 (0.77- 1.17) | 0.642 | 0.88 (0.71- 1.10) | 0.287 | 0.85 (0.68- 1.06) | 0.161 |
|  | Stage 1 HTN | 1.19 (1.01- 1.39) | **0.032** | 1.13 (0.96- 1.33) | 0.141 | 1.08 (0.91- 1.28) | 0.357 |
|  | Stage 2 HTN | 1.19 (1.02- 1.38) | **0.020** | 1.06 (0.90- 1.24) | 0.487 | 0.96 (0.80- 1.14) | 0.674 |
| C5DC | Elevated BP | 0.89 (0.73- 1.10) | 0.310 | 0.84 (0.67- 1.05) | 0.139 | 0.85 (0.67- 1.06) | 0.159 |
|  | Stage 1 HTN | 1.07 (0.92- 1.26) | 0.349 | 1.03 (0.87- 1.22) | 0.701 | 1.02 (0.85- 1.22) | 0.777 |
|  | Stage 2 HTN | 1.23 (1.06- 1.43) | **0.006** | 1.13 (0.95- 1.33) | 0.157 | 1.09 (0.91- 1.31) | 0.302 |
| C6 | Elevated BP | 0.90 (0.73- 1.12) | 0.383 | 0.87 (0.70- 1.08) | 0.220 | 0.87 (0.70- 1.08) | 0.227 |
|  | Stage 1 HTN | 1.09 (0.92- 1.28) | 0.290 | 1.05 (0.89- 1.23) | 0.520 | 1.02 (0.87- 1.20) | 0.762 |
|  | Stage 2 HTN | 1.20 (1.03- 1.39) | **0.015** | 1.11 (0.95- 1.31) | 0.173 | 1.07 (0.91- 1.27) | 0.384 |
| C8 | Elevated BP | 0.93 (0.75- 1.16) | 0.541 | 0.90 (0.72- 1.12) | 0.360 | 0.91 (0.73- 1.14) | 0.454 |
|  | Stage 1 HTN | 1.13 (0.96- 1.32) | 0.138 | 1.09 (0.92- 1.27) | 0.295 | 1.06 (0.90- 1.25) | 0.439 |
|  | Stage 2 HTN | 1.22 (1.05- 1.42) | **0.008** | 1.15 (0.98- 1.34) | 0.084 | 1.11 (0.94- 1.32) | 0.200 |
| C8:1 | Elevated BP | 0.85 (0.69- 1.05) | 0.143 | 0.80 (0.65- 0.99) | **0.047** | 0.82 (0.66- 1.02) | 0.076 |
|  | Stage 1 HTN | 1.12 (0.96- 1.32) | 0.138 | 1.09 (0.92- 1.28) | 0.292 | 1.09 (0.92- 1.29) | 0.311 |
|  | Stage 2 HTN | 1.32 (1.13- 1.53) | **<0.001** | 1.18 (1.00- 1.39) | **0.038** | 1.18 (0.99- 1.40) | 0.057 |
| C10 | Elevated BP | 0.93 (0.75- 1.15) | 0.508 | 0.90 (0.73- 1.12) | 0.356 | 0.92 (0.74- 1.14) | 0.453 |
|  | Stage 1 HTN | 1.13 (0.96- 1.32) | 0.136 | 1.09 (0.93- 1.28) | 0.262 | 1.07 (0.90- 1.26) | 0.407 |
|  | Stage 2 HTN | 1.27 (1.09- 1.48) | **0.002** | 1.20 (1.02- 1.41) | **0.021** | 1.16 (0.98- 1.38) | 0.076 |
| C10:1 | Elevated BP | 0.96 (0.78- 1.19) | 0.743 | 0.94 (0.76- 1.17) | 0.625 | 0.97 (0.78- 1.21) | 0.814 |
|  | Stage 1 HTN | 1.14 (0.97- 1.35) | 0.091 | 1.11 (0.95- 1.31) | 0.175 | 1.10 (0.93- 1.30) | 0.240 |
|  | Stage 2 HTN | 1.25 (1.07- 1.45) | **0.004** | 1.20 (1.02- 1.41) | **0.021** | 1.18 (0.99- 1.40) | 0.053 |
| C12 | Elevated BP | 0.87 (0.70- 1.07) | 0.204 | 0.82 (0.66- 1.02) | 0.088 | 0.84 (0.67- 1.04) | 0.116 |
|  | Stage 1 HTN | 1.09 (0.93- 1.28) | 0.276 | 1.04 (0.89- 1.23) | 0.563 | 1.04 (0.88- 1.22) | 0.639 |
|  | Stage 2 HTN | 1.30 (1.12- 1.52) | **<0.001** | 1.19 (1.01- 1.40) | **0.029** | 1.18 (1.00- 1.40) | **0.048** |
| C14 | Elevated BP | 0.86 (0.70- 1.07) | 0.193 | 0.80 (0.64- 0.99) | **0.049** | 0.81 (0.65- 1.01) | 0.068 |
|  | Stage 1 HTN | 1.05 (0.89- 1.23) | 0.555 | 1.00 (0.85- 1.18) | 0.939 | 0.99 (0.84- 1.17) | 0.973 |
|  | Stage 2 HTN | 1.33 (1.15- 1.55) | **<0.001** | 1.16 (0.98- 1.36) | 0.071 | 1.14 (0.96- 1.35) | 0.120 |
| C14:1 | Elevated BP | 0.86 (0.70- 1.06) | 0.181 | 0.82 (0.66- 1.02) | 0.082 | 0.85 (0.69- 1.06) | 0.164 |
|  | Stage 1 HTN | 1.00 (0.85- 1.18) | 0.916 | 0.98 (0.83- 1.15) | 0.840 | 0.99 (0.84- 1.18) | 0.994 |
|  | Stage 2 HTN | 1.29 (1.11- 1.49) | **0.001** | 1.17 (0.99- 1.37) | 0.053 | 1.20 (1.01- 1.42) | **0.030** |
| C14:2 | Elevated BP | 0.89 (0.72- 1.10) | 0.288 | 0.88 (0.71- 1.09) | 0.252 | 0.91 (0.73- 1.12) | 0.391 |
|  | Stage 1 HTN | 1.07 (0.91- 1.25) | 0.394 | 1.05 (0.89- 1.23) | 0.538 | 1.06 (0.90- 1.26) | 0.437 |
|  | Stage 2 HTN | 1.21 (1.04- 1.40) | **0.011** | 1.17 (1.00- 1.38) | **0.047** | 1.19 (1.01- 1.41) | **0.036** |
| C14OH | Elevated BP | 1.03 (0.84- 1.27) | 0.736 | 0.94 (0.76- 1.17) | 0.625 | 0.95 (0.76- 1.19) | 0.695 |
|  | Stage 1 HTN | 1.13 (0.96- 1.33) | 0.117 | 1.09 (0.92- 1.29) | 0.295 | 1.07 (0.90- 1.27) | 0.409 |
|  | Stage 2 HTN | 1.42 (1.22- 1.65) | **<0.001** | 1.18 (1.00- 1.40) | **0.041** | 1.14 (0.96- 1.36) | 0.128 |
| C16 | Elevated BP | 0.97 (0.79- 1.20) | 0.811 | 0.89 (0.72- 1.11) | 0.330 | 0.87 (0.70- 1.09) | 0.240 |
|  | Stage 1 HTN | 1.03 (0.88- 1.21) | 0.674 | 0.97 (0.82- 1.15) | 0.773 | 0.91 (0.77- 1.08) | 0.299 |
|  | Stage 2 HTN | 1.36 (1.17- 1.59) | **<0.001** | 1.17 (1.00- 1.38) | 0.050 | 1.10 (0.93- 1.31) | 0.257 |
| C16OH | Elevated BP | 0.82 (0.66- 1.01) | 0.069 | 0.73 (0.58- 0.92) | **0.007** | 0.72 (0.58- 0.91) | **0.005** |
|  | Stage 1 HTN | 1.04 (0.88- 1.22) | 0.640 | 0.99 (0.83- 1.17) | 0.937 | 0.94 (0.79- 1.12) | 0.540 |
|  | Stage 2 HTN | 1.35 (1.16- 1.57) | **<0.001** | 1.12 (0.95- 1.33) | 0.158 | 1.06 (0.89- 1.26) | 0.480 |
| C16:1OH | Elevated BP | 0.85 (0.69- 1.05) | 0.152 | 0.76 (0.61-0.95) | **0.020** | 0.77 (0.62- 0.96) | **0.025** |
|  | Stage 1 HTN | 1.01 (0.86- 1.19) | 0.856 | 0.96 (0.81- 1.14) | .0701 | 0.96 (0.81- 1.13) | 0.636 |
|  | Stage 2 HTN | 1.26 (1.08- 1.46) | **0.002** | 1.04 (0.88- 1.23) | 0.582 | 1.04 (0.88- 1.24) | 0.610 |
| C16:1 | Elevated BP | 0.98 (0.79- 1.20) | 0.853 | 0.88 (0.71- 1.09) | 0.260 | 0.91 (0.73- 1.13) | 0.396 |
|  | Stage 1 HTN | 0.97 (0.82- 1.14) | 0.719 | 0.93 (0.78- 1.09) | 0.393 | 0.93 (0.78- 1.10) | 0.437 |
|  | Stage 2 HTN | 1.37 (1.18- 1.60) | **<0.001** | 1.12 (0.95- 1.33) | 0.148 | 1.14 (0.95- 1.35) | 0.137 |
| C18 | Elevated BP | 0.90 (0.73- 1.11) | 0.350 | 0.84 (0.68- 1.05) | 0.144 | 0.79 (0.63- 1.00) | 0.055 |
|  | Stage 1 HTN | 0.99 (0.84- 1.16) | 0.955 | 0.95 (0.80- 1.12) | 0.575 | 0.87 (0.73- 1.04) | 0.147 |
|  | Stage 2 HTN | 1.21 (1.05- 1.41) | **0.009** | 1.08 (0.91- 1.27) | 0.352 | 0.99 (0.83- 1.18) | 0.958 |
| C18:1 | Elevated BP | 0.96 (0.78- 1.19) | 0.767 | 0.90 (0.73- 1.12) | 0.373 | 0.92 (0.74- 1.15) | 0.490 |
|  | Stage 1 HTN | 0.94 (0.80- 1.10) | 0.469 | 0.91 (0.77- 1.07) | 0.264 | 0.91 (0.77- 1.08) | 0.299 |
|  | Stage 2 HTN | 1.26 (1.09- 1.47) | **0.002** | 1.09 (0.93- 1.29) | 0.255 | 1.11 (0.93- 1.31) | 0.234 |
| C18OH | Elevated BP | 1.01 (0.82- 1.25) | 0.870 | 0.93 (0.74- 1.15) | 0.517 | 0.87 (0.70- 1.09) | 0.241 |
|  | Stage 1 HTN | 1.18 (1.00- 1.38) | **0.045** | 1.12 (0.95- 1.32) | 0.175 | 1.04 (0.87- 1.23) | 0.630 |
|  | Stage 2 HTN | 1.29 (1.11- 1.51) | **0.001** | 1.08 (0.91- 1.27) | 0.348 | 0.96 (0.80- 1.14) | 0.657 |
| C18:1OH | Elevated BP | 0.90 (0.72- 1.11) | 0.330 | 0.82 (0.66- 1.03) | 0.092 | 0.85 (0.68- 1.06) | 0.159 |
|  | Stage 1 HTN | 0.98 (0.83- 1.15) | 0.820 | 0.94 (0.80- 1.12) | 0.538 | 0.95 (0.80- 1.13) | 0.576 |
|  | Stage 2 HTN | 1.25 (1.08- 1.45) | **0.003** | 1.05 (0.89- 1.24) | 0.500 | 1.05 (0.89- 1.25) | 0.514 |
| C18:2OH | Elevated BP | 1.10 (0.90- 1.36) | 0.334 | 1.07 (0.87- 1.32) | 0.488 | 0.99 (0.79- 1.23) | 0.949 |
|  | Stage 1 HTN | 1.06 (0.90- 1.24) | 0.478 | 1.03 (0.87- 1.21) | 0.709 | 0.94 (0.79- 1.11) | 0.487 |
|  | Stage 2 HTN | 1.20 (1.03- 1.40) | **0.016** | 1.13 (0.96- 1.33) | 0.122 | 0.99 (0.83- 1.17) | 0.937 |
| Alanine | Elevated BP | 1.15 (0.94- 1.40) | 0.172 | 1.06 (0.86- 1.30) | 0.574 | 0.95 (0.75- 1.19) | 0.664 |
|  | Stage 1 HTN | 1.36 (1.16- 1.60) | **<0.001** | 1.26 (1.07- 1.49) | **0.005** | 1.11 (0.93- 1.32) | 0.245 |
|  | Stage 2 HTN | 1.39 (1.20- 1.62) | **<0.001** | 1.20 (1.02- 1.42) | **0.021** | 1.02 (0.85- 1.22) | 0.800 |
| Aspartic Acid | Elevated BP | 0.83 (0.68- 1.02) | 0.091 | 0.87 (0.70- 1.07) | 0.203 | 0.84 (0.68- 1.04) | 0.115 |
|  | Stage 1 HTN | 0.91 (0.77- 1.06) | 0.251 | 0.92 (0.78- 1.09) | 0.351 | 0.89 (0.75- 1.06) | 0.205 |
|  | Stage 2 HTN | 0.84 (0.72- 0.97) | **0.022** | 0.89 (0.76- 1.04) | 0.170 | 0.87 (0.74- 1.04) | 0.134 |
| Glutamic Acid | Elevated BP | 1.14 (0.93- 1.40) | 0.189 | 1.09 (0.88- 1.35) | 0.388 | 1.04 (0.83- 1.30) | 0.700 |
|  | Stage 1 HTN | 1.21 (1.03- 1.42) | **0.016** | 1.13 (0.96- 1.33) | 0.128 | 1.07 (0.90- 1.27) | 0.417 |
|  | Stage 2 HTN | 1.17 (1.01- 1.36) | **0.035** | 1.08 (0.92- 1.27) | 0.327 | 0.98 (0.83- 1.17) | 0.900 |
| Leucine | Elevated BP | 1.21 (0.99- 1.49) | 0.059 | 1.22 (0.97- 1.54) | 0.079 | 1.10 (0.85- 1.42) | 0.437 |
|  | Stage 1 HTN | 1.25 (1.06- 1.47) | **0.006** | 1.16 (0.97- 1.39) | 0.091 | 0.98 (0.80- 1.20) | 0.854 |
|  | Stage 2 HTN | 1.18 (1.02- 1.37) | **0.024** | 1.13 (0.95- 1.35) | 0.147 | 0.92 (0.75- 1.12) | 0.415 |
| Methionine | Elevated BP | 0.98 (0.79- 1.20) | 0.846 | 1.01 (0.81- 1.26) | 0.882 | 1.00 (0.81- 1.25) | 0.942 |
|  | Stage 1 HTN | 0.97 (0.83- 1.14) | 0.780 | 0.94 (0.80- 1.12) | 0.541 | 0.92 (0.78- 1.09) | 0.380 |
|  | Stage 2 HTN | 0.93 (0.81- 1.08) | 0.402 | 1.00 (0.84- 1.17) | 0.999 | 1.00 (0.84- 1.19) | 0.942 |
| Phenylalanine | Elevated BP | 1.08 (0.88- 1.33) | 0.441 | 1.01 (0.81- 1.25) | 0.904 | 0.95 (0.76- 1.19) | 0.674 |
|  | Stage 1 HTN | 1.08 (0.92- 1.26) | 0.344 | 0.99 (0.84- 1.17) | 0.951 | 0.93 (0.78- 1.11) | 0.465 |
|  | Stage 2 HTN | 1.19 (1.03- 1.39) | **0.018** | 1.05 (0.89- 1.24) | 0.520 | 1.02 (0.85- 1.22) | 0.815 |
| Tyrosine | Elevated BP | 1.19 (0.96- 1.46) | 0.096 | 1.10 (0.88- 1.36) | 0.389 | 1.02 (0.82- 1.29) | 0.807 |
|  | Stage 1 HTN | 1.12 (0.96- 1.31) | 0.147 | 1.00 (0.85- 1.19) | 0.933 | 0.90 (0.75- 1.08) | 0.267 |
|  | Stage 2 HTN | 1.17 (1.01- 1.35) | **0.037** | 0.99 (0.84- 1.17) | 0.976 | 0.90 (0.75- 1.07) | 0.260 |
| Valine | Elevated BP | 1.27 (1.03- 1.56) | **0.021** | 1.25 (1.00- 1.57) | **0.049** | 1.12 (0.87- 1.45) | 0.359 |
|  | Stage 1 HTN | 1.30 (1.11- 1.53) | **0.001** | 1.20 (1.01- 1.44) | **0.033** | 1.00 (0.81- 1.22) | 0.976 |
|  | Stage 2 HTN | 1.29 (1.11- 1.50) | **0.001** | 1.20 (1.01- 1.43) | **0.033** | 0.95 (0.78- 1.17) | 0.676 |
| Arginine | Elevated BP | 1.07 (0.87- 1.31) | 0.515 | 1.09 (0.88- 1.34) | 0.403 | 1.08 (0.87- 1.34) | 0.471 |
|  | Stage 1 HTN | 1.10 (0.94- 1.29) | 0.226 | 1.10 (0.94- 1.29) | 0.225 | 1.06 (0.90- 1.25) | 0.456 |
|  | Stage 2 HTN | 0.94 (0.81- 1.09) | 0.437 | 0.99 (0.84- 1.15) | 0.916 | 0.97 (0.82- 1.15) | 0.769 |
| Citrulline | Elevated BP | 0.99 (0.81- 1.21) | 0.959 | 0.93 (0.74- 1.16) | 0.550 | 0.93 (0.73- 1.17) | 0.556 |
|  | Stage 1 HTN | 1.03 (0.88- 1.20) | 0.686 | 0.99 (0.83- 1.19) | 0.982 | 0.99 (0.82- 1.19) | 0.917 |
|  | Stage 2 HTN | 1.04 (0.90- 1.21) | 0.521 | 0.91 (0.77- 1.08) | 0.306 | 0.88 (0.73- 1.06) | 0.186 |
| Glycine | Elevated BP | 0.99 (0.81- 1.21) | 0.959 | 1.01 (0.82- 1.24) | 0.907 | 1.07 (0.86- 1.34) | 0.497 |
|  | Stage 1 HTN | 0.82 (0.70- 0.96) | **0.016** | 0.85 (0.72- 1.00) | 0.057 | 0.92 (0.77- 1.09) | 0.356 |
|  | Stage 2 HTN | 0.73 (0.63- 0.85) | **<0.001** | 0.75 (0.64- 0.88) | **0.001** | 0.81 (0.68- 0.96) | **0.020** |
| Ornithine | Elevated BP | 1.07 (0.87- 1.31) | 0.512 | 1.00 (0.81- 1.24) | 0.942 | 0.98 (0.78- 1.22) | 0.866 |
|  | Stage 1 HTN | 1.09 (0.93- 1.28) | 0.248 | 1.03 (0.88- 1.22) | 0.641 | 0.99 (0.83- 1.17) | 0.932 |
|  | Stage 2 HTN | 1.15 (0.99- 1.34) | 0.054 | 1.03 (0.88- 1.21) | 0.672 | 0.99 (0.83- 1.17) | 0.910 |
| Proline | Elevated BP | 1.01 (0.82- 1.24) | 0.919 | 0.98 (0.79- 1.22) | 0.899 | 0.95 (0.76- 1.19) | 0.673 |
|  | Stage 1 HTN | 1.24 (1.06- 1.46) | **0.007** | 1.18 (1.00- 1.40) | **0.047** | 1.13 (0.95- 1.34) | 0.161 |
|  | Stage 2 HTN | 1.18 (1.01- 1.37) | **0.030** | 1.11 (0.94- 1.31) | 0.199 | 1.03 (0.87- 1.24) | 0.669 |
| Threonine | Elevated BP | 0.98 (0.80- 1.21) | 0.904 | 1.02 (0.83- 1.26) | 0.808 | 1.03 (0.83- 1.28) | 0.744 |
|  | Stage 1 HTN | 0.98 (0.83- 1.15) | 0.831 | 0.97 (0.82- 1.14) | 0.759 | 0.99 (0.84- 1.17) | 0.940 |
|  | Stage 2 HTN | 0.85 (0.73- 0.99) | **0.040** | 0.92 (0.78- 1.08) | 0.325 | 0.94 (0.79- 1.11) | 0.499 |
| Serine | Elevated BP | 0.90 (0.73- 1.11) | 0.364 | 0.95 (0.77- 1.17) | 0.664 | 1.03 (0.82- 1.28) | 0.788 |
|  | Stage 1 HTN | 0.84 (0.71- 0.98) | **0.033** | 0.88 (0.74- 1.03) | 0.130 | 0.98 (0.82- 1.16) | 0.827 |
|  | Stage 2 HTN | 0.67 (0.58- 0.78) | **<0.001** | 0.75 (0.63- 0.88) | **0.001** | 0.86 (0.72- 1.03) | 0.122 |
| Histidine | Elevated BP | 0.90 (0.74- 1.11) | 0.364 | 0.96 (0.78- 1.18) | 0.737 | 0.95 (0.76- 1.18) | 0.643 |
|  | Stage 1 HTN | 0.95 (0.81- 1.12) | 0.574 | 0.98 (0.83- 1.16) | 0.871 | 0.99 (0.84- 1.18) | 0.956 |
|  | Stage 2 HTN | 0.84 (0.72- 0.98) | **0.026** | 0.96 (0.82- 1.12) | 0.641 | 1.00 (0.84- 1.19) | 0.931 |
| Lysine | Elevated BP | 1.06 (0.86- 1.30) | 0.567 | 1.07 (0.86- 1.31) | 0.526 | 1.06 (0.85- 1.31) | 0.589 |
|  | Stage 1 HTN | 0.99 (0.84- 1.16) | 0.910 | 0.99 (0.85- 1.17) | 0.979 | 0.99 (0.84- 1.17) | 0.944 |
|  | Stage 2 HTN | 1.01 (0.87- 1.17) | 0.874 | 1.01 (0.86- 1.19) | 0.827 | 1.07 (0.90- 1.26) | 0.428 |
| Tryptophan | Elevated BP | 1.22 (0.99- 1.50) | 0.056 | 1.29 (1.03- 1.61) | **0.025** | 1.24 (0.98- 1.57) | 0.063 |
|  | Stage 1 HTN | 1.10 (0.94- 1.29) | 0.231 | 1.07 (0.90- 1.26) | 0.425 | 0.99 (0.83- 1.19) | 0.960 |
|  | Stage 2 HTN | 0.99 (0.85- 1.15) | 0.943 | 1.08 (0.91- 1.27) | 0.346 | 1.01 (0.84- 1.21) | 0.867 |
| Asparagine | Elevated BP | 0.82 (0.66- 1.00) | 0.061 | 0.86 (0.69- 1.06) | 0.163 | 0.87 (0.70- 1.08) | 0.220 |
|  | Stage 1 HTN | 0.83 (0.70- 0.97) | **0.025** | 0.85 (0.72- 1.00) | 0.062 | 0.90 (0.75- 1.06) | 0.226 |
|  | Stage 2 HTN | 0.86 (0.74- 1.00) | 0.057 | 0.94 (0.80- 1.11) | 0.485 | 1.04 (0.87- 1.24) | 0.650 |
| Glutamine | Elevated BP | 0.97 (0.79- 1.19) | 0.824 | 0.97 (0.78- 1.19) | 0.785 | 0.96 (0.78- 1.19) | 0.738 |
|  | Stage 1 HTN | 0.98 (0.84- 1.15) | 0.843 | 0.98 (0.83- 1.15) | 0.836 | 0.98 (0.83- 1.16) | 0.868 |
|  | Stage 2 HTN | 0.99 (0.85- 1.14) | 0.905 | 0.97 (0.83- 1.14) | 0.750 | 1.04 (0.88- 1.23) | 0.641 |

*Model 1 is adjusted for age, sex and BMI.

**Model 2 is adjusted for age, sex, BMI, total cholesterol, triglyceride, HDL cholesterol, FPG, oral glucose-lowering drugs use, statin use and antihypertensive drugs use.

C0: Free carnitine, C2: Acetylcarnitine, C3: Propionylcarnitine, C3DC: Malonylcarnitine, C4: Butyrylcarnitine, C4DC: Methylmalonyl-/succinylcarn, C4OH: 3-OH-iso-/Butyrylcarnitine, C5: isovalerylcarnitine, C5:1: Tiglylcarnitine, C5OH: 3-OH-Isovalerylcarnitine, C5DC: Glutarylcarnitine, C6: Hexanoylcarnitine, C8: Octanoylcarnitine, C8:1: Octenoylcarnitine, C10: Decanoylcarnitine, C10:1: Decenoylcarnitine, C12: Dodecanoylcarnitine, C14: Tetradecanoylcarnitine, C14:1: Tetradecenoylcarnitine, C14:2: Tetradecadienoylcarnitine, C14OH: 3-OH-Tetradecanoylcarnitine, C16: Hexadecanoylcarnitine, C16OH: 3-OH-hexadecanoylcarnitine, C16:1OH: 3-OH-Hexadecenoylcarnitine, C16:1: Hexadecenoylcarnitine, C18: Octadecanoylcarnitine, C18:1: Octadecenoylcarnitine, C18OH: 3-OH-Octadecanoylcarnitine, C18:1OH: 3-OH-Octadecenoylcarnitine, C18:2: Octadecadienoylcarnitine, C18:2OH: 3-OH-octadecadienoylcarn
