# Supplementary material for: Association of plasma acylcarnitines and amino acids with hypertension: A nationwide metabolomics study
Source: PLoS One. 2023 Jan 17;18(1):e0279835. doi: 10.1371/journal.pone.0279835 (PMC9844860; doi:10.1371/journal.pone.0279835)
Supplement: S4 Table — (DOCX) [file pone.0279835.s004.docx]

S4 Table. The formula of 11 factors applying for factor analysis

| Components | calculation |
| --- | --- |
| Factor 1 | (0.788 * C16:1OH) + (0.772 * C18:1) + (0.76 * C18) + (0.754 * C18:1OH) + (0.745 * C16) + (0.738 * C16OH) + (0.704 * C14) + (0.683 * C16:1) + (0.665 * C18OH) + (0.645 * C14OH) |
| Factor 2 | (0.926 * C8) + (0.924 * C10:1) + (0.922 * C10) + (0.804 * C14:2) + (0.756 * C12) + (0.754 * C14:1) + (0.740 * C6) + (0.602 * C5DC) + (0.431 * C4) + (0.476 * C8:1) |
| Factor 3 | (0.787 * Tyrosin) + (0.773 * Methionine) + (0.737 * Leucine) + (0.712 * Tryptophan) + (0.656 * Phenylalanine) + (0.503 * Threonine) |
| Factor 4 | (0.909 * Lysine) + (0.886 * Glutamine) + (0.687 * Histidine) + (0.662 * Asparagine) |
| Factor 5 | (0.861 * C5:1) + (0.747 * C5OH) + (0.561 * C5) + (0.472 * C3DC) |
| Factor 6 | (0.446 * C5) + (0.645 * C3) + (0.638 * C0) + (0.485 * C4) + (0.428 * Ornitine) |
| Factor 7 | (0.814 * Glycine) + (0.708 * Serine) |
| Factor 8 | (0.643 * Proline) + (0.632 * Alanine) |
| Factor 9 | (0.593 * C4OH) + (0.560 * C2) + (0.555 * C8:1) |
| Factor 10 | (0.649 * C18:2OH) + (0.645 * Glutamic acid) + (0.468 * Aspartic acid) |
| Factor 11 | (0.759 * Citrulline) + (0.604 * Arginine) |
