# Supplementary material for: Association of plasma acylcarnitines and amino acids with hypertension: A nationwide metabolomics study
Source: PLoS One. 2023 Jan 17;18(1):e0279835. doi: 10.1371/journal.pone.0279835 (PMC9844860; doi:10.1371/journal.pone.0279835)
Supplement: Attachment — Submitted filename: PONE-D-21-34134_Review.docx [file pone.0279835.s005.docx]

# PONE-D-21-34134

Thank you for the opportunity to review the manuscript by Razi et al. In this interesting research article, link between plasma amino acids and acylcarnitines with different blood pressure categories ranging from normal BP to elevated to the different stages of hypertension were investigated. Two metabolite clusters were identified with factor analyses. The first factor consisting of various short- and medium chain acylcarnitines was associated with an increased odds of having stage 2 hypertension, while a second factor consisting of two amino acids, namely glycine and serine was associated with a lower odds of have stage 2 hypertension. I do have the following comments and suggestions:

- Overall, the manuscript would benefit greatly from professional language and technical editing.
- Abstract: Line 27: What is meant with different stages of blood pressure. This should be phrased so that it is clear that you divided your groups based on different blood pressure cut points to classify normal, high and hypertensive stages.
- Abstract: Which criteria did you use for blood pressure classification
- Abstract: Line 36-37: The study design and statistical approach does not allow you to determine cause and effect. Therefore, you should rephrase: “to estimate the effect of metabolites on the risk of hypertension.”
- Abstract: Perhaps indicate the confounders you adjusted for.
- Abstract: Be more specific regarding the indexes (or factors) you identified. It is stated in a much clearer fashion in the conclusion of the article.
- Introduction: Line 53: “which shows a significant growth trend.” Can perhaps be rephrased to indicate that there was an upward trajectory.”
- Introduction: Line 57: “….it might be ignored.” It is not clear what is meant here? What might be ignored?
- Introduction: Line 60-62: The mention of biomarkers not related to metabolomics per se is not applicable here. Perhaps you should rather refer to what was found in previous metabolomics papers?
- Introduction: Line 61-62: “….no parameters have been confirmed for diagnosing and predicting hypertension.” This statement is simply not true as there are many factors previously reported on (including biomarkers) that can be used for the prediction of hypertension. Perhaps I misunderstood you. Please clarify.
- Introduction: Line 66: What is meant with metabolomics is a practical method? It requires expensive equipment and trained staff to perform these analyses?
- Introduction: Line 68: The reference to organic acids is quite broad as there are many organic acids that form part of multiple metabolic pathways?
- Methods: Line 83: Please specify the device used to measure BP
- Methods: For both the biochemical measurements and the metabolomics analysis the coefficient of variation should be reported.
- Methods: What type of QCs did you incorporate in your metabolomics analyses. Was it done in batches? How did you correct for batch effects?
- Methods: Line 101: Reference is made to internal standards. What was used? What concentrations?
- Methods: What is the level of certainty of the identification of your metabolites? Did you compare it with commercially available standards? How did you quantify the levels of your metabolites?
- Methods: Line 121: Adjustments were made for the lipid profile? Did you include all measures as part of the lipid profile? Or only HDL? Be specific.
- Methods: Line 121: How did you decide on FPG to adjust for instead of HbA1c?
- Results: Line 127-128: Instead of indication that each group consisted of almost half females, rather indicate that there was an equal sex distribution.
- Results: Line 131: I am not sure where the (p≤0.026) comes from as all the p-trend values indicated in Table 1 (with the exception of statin use) are 0.001? Did you also compare the groups in post-hoc analyses? If you did, it should be indicated in Table 1.
- Did you adjust for statin use or the use of other medication? Perhaps that should be included as sensitivity analyses?
- What was the rationale for inter-correlating the metabolites (Fig1). As this was not part of your original aim?
- Table 2: It is not clear what exactly you are reporting. Did you compare all groups? Or did you compare all groups with the control group? I think it may be worthwhile to do the latter if you did not. Also, to just indicate that there are differences without indicating if it was higher/lower does not say much?
- Discussion: Line 205 & 246: I think the use of the work “upregulated” is not appropriate here. Rather say that the abundance of acylcarnitines were higher. Normally you would use the term upregulated for an enzyme and in this case if acylcarnitines are accumulated it would suggest that the beta-oxidation enzymes are not functioning optimally?
- Discussion: Line 205-207: It is indicated that beta-oxidation may not be functionally optimally. Can you speculate as to why this is the case?
- Discussion: Line 254: It is indicated that plasma is the best sample choice. It should be considered that urine can also be used and that urine is a lot less invasive to collect.
- Conclusion: Line 261: “….found the unique metabolite profile of plasma” This phrase in unclear. Also the phrase “….different stages of blood pressure” is not clear and should be clarified.
