# Supplementary material for: Association of plasma acylcarnitines and amino acids with hypertension: A nationwide metabolomics study
Source: PLoS One. 2023 Jan 17;18(1):e0279835. doi: 10.1371/journal.pone.0279835 (PMC9844860; doi:10.1371/journal.pone.0279835)
Supplement: Attachment — Submitted filename: Response to Reviewers.docx [file pone.0279835.s006.docx]

Response to Reviewers

Journal Requirements:

When submitting your revision, we need you to address these additional requirements.

1. Please ensure that your manuscript meets PLOS ONE's style requirements, including those for file naming. The PLOS ONE style templates can be found at
   <https://journals.plos.org/plosone/s/file?id=wjVg/PLOSOne_formatting_sample_main_body.pdf> and 
   <https://journals.plos.org/plosone/s/file?id=ba62/PLOSOne_formatting_sample_title_authors_affiliations.pdf>

Done.

2. In your Data Availability statement, you have not specified where the minimal data set underlying the results described in your manuscript can be found. PLOS defines a study's minimal data set as the underlying data used to reach the conclusions drawn in the manuscript and any additional data required to replicate the reported study findings in their entirety. All PLOS journals require that the minimal data set be made fully available. For more information about our data policy, please see <http://journals.plos.org/plosone/s/data-availability>.

Upon re-submitting your revised manuscript, please upload your study’s minimal underlying data set as either Supporting Information files or to a stable, public repository and include the relevant URLs, DOIs, or accession numbers within your revised cover letter. For a list of acceptable repositories, please see <http://journals.plos.org/plosone/s/data-availability#loc-recommended-repositories>. Any potentially identifying patient information must be fully anonymized.

Important: If there are ethical or legal restrictions to sharing your data publicly, please explain these restrictions in detail. Please see our guidelines for more information on what we consider unacceptable restrictions to publicly sharing data: <http://journals.plos.org/plosone/s/data-availability#loc-unacceptable-data-access-restrictions>. Note that it is not acceptable for the authors to be the sole named individuals responsible for ensuring data access.

We will update your Data Availability statement to reflect the information you provide in your cover letter.

Done. All relevant data are within the manuscript and its Supporting Information files.

3. PLOS requires an ORCID iD for the corresponding author in Editorial Manager on papers submitted after December 6th, 2016. Please ensure that you have an ORCID iD and that it is validated in Editorial Manager. To do this, go to ‘Update my Information’ (in the upper left-hand corner of the main menu), and click on the Fetch/Validate link next to the ORCID field. This will take you to the ORCID site and allow you to create a new iD or authenticate a pre-existing iD in Editorial Manager. Please see the following video for instructions on linking an ORCID iD to your Editorial Manager account: <https://www.youtube.com/watch?v=_xcclfuvtxQ>

Done.

4. Please upload a new copy of Figure 1 as the detail is not clear. Please follow the link for more information:<https://blogs.plos.org/plos/2019/06/looking-good-tips-for-creating-your-plos-figures-graphics/>" <https://blogs.plos.org/plos/2019/06/looking-good-tips-for-creating-your-plos-figures-graphics/>

Done.

5. Please include captions for your Supporting Information files at the end of your manuscript, and update any in-text citations to match accordingly. Please see our Supporting Information guidelines for more information: <http://journals.plos.org/plosone/s/supporting-information>.

Done. Thanks for your precise comment.


[Note: HTML markup is below. Please do not edit.]

Reviewers' comments:

Reviewer #1: This is a cross sectional study examining serum metabolomics profile in subjects with varying levels of hypertension in Iran. 1200 subjects were selected from a national survey. The subjects were stratified according to blood pressure level. It was not clear if any of the subjects were taking medication for blood pressure. Serum was obtained and subjected to metabolomics analysis to measure acylcarnitines and amino acids. Appropriate statistical handling was performed including regression as well as principal component analysis to look for association between serum metabolites and blood pressure levels. After several adjustments for metabolic disease risk factors several acylcarnitine species were associated with higher odds of elevated blood pressure and glycine was associated with lower odds of elevated blood pressure. Subsequently, principal component analysis was applied to look for factors which risk of increased blood pressure. After adjustment a factor containing mostly medium and short chain acylcarnitines was associated with higher risk while factors containing glycine and serine and another containing ornithine and short chain acyl-carnitines were associated with lower risk of elevated blood pressure. The discussion reviews the finding in the context of what is known about metabolomics and hypertension as well as proposing possible biologic mechanisms to explain their findings.
Overall the study was well conducted with a large number of study subjects. The results add to our understanding of serum metabolome in subjects with elevated blood pressure.

One minor comment: I would have liked to know if any of the subjects in the study were on anti-hypertensive medications.

Done. Thank you so much for your appropriate comment. The number of subjects that were on anti-hypertensive medication in each group were provided in Table 1 under the name of “Antihypertensive drugs, n (%)”. We also described the number of subjects that were on anti-hypertensive medications in the result section of the revised manuscript.

Reviewer #2: Thank you for the opportunity to review the manuscript by Razi et al. In this interesting research article, link between plasma amino acids and acylcarnitines with different blood pressure categories ranging from normal BP to elevated to the different stages of hypertension were investigated. Two metabolite clusters were identified with factor analyses. The first factor consisting of various short- and medium chain acylcarnitines was associated with an increased odds of having stage 2 hypertension, while a second factor consisting of two amino acids, namely glycine and serine was associated with a lower odds of have stage 2 hypertension. I do have the following comments and suggestions:
• Overall, the manuscript would benefit greatly from professional language and technical editing.

Done. Thanks for your appropriate comment. We revised the manuscript in the sight of professional language and technical editing.

• Abstract: Line 27: What is meant with different stages of blood pressure. This should be phrased so that it is clear that you divided your groups based on different blood pressure cut points to classify normal, high and hypertensive stages.

Done, Thanks for your accurate comment. We rephrased this sentence in the revised manuscript and it is now clear that we divided the study population into 4 groups of normal blood pressure, elevated blood pressure, stage 1 and stage 2 hypertension.

• Abstract: Which criteria did you use for blood pressure classification

Done. Thanks for your appropriate comment. We declared in the abstract section of the revised manuscript that we used ACC/AHA hypertension guidelines to classified our study population.

• Abstract: Line 36-37: The study design and statistical approach does not allow you to determine cause and effect. Therefore, you should rephrase: “to estimate the effect of metabolites on the risk of hypertension.”

Done. You are absolutely correct. We rephrased this sentence in the revised manuscript. We used the association between two variables instead of causality.

• Abstract: Perhaps indicate the confounders you adjusted for.

Done. Thanks for your comment. We indicated the confounders we adjusted for in the abstract section of the revised manuscript.

• Abstract: Be more specific regarding the indexes (or factors) you identified. It is stated in a much clearer fashion in the conclusion of the article.

Done. You are right. Thanks for your appropriate comment We specified the factors in the result of abstract section in the revised manuscript.

• Introduction: Line 53: “which shows a significant growth trend.” Can perhaps be rephrased to indicate that there was an upward trajectory.”

Done. Thank you for your comment. We rephrased it in the revised manuscript.

• Introduction: Line 57: “….it might be ignored.” It is not clear what is meant here? What might be ignored?

Done. Thanks for your appropriate comment. We rephrased it in the revised manuscript. We meant that HTN may not be diagnosed due to variation in blood pressure, observer bias, and using nonstandard devices.

• Introduction: Line 60-62: The mention of biomarkers not related to metabolomics per se is not applicable here. Perhaps you should rather refer to what was found in previous metabolomics papers?

Done. You are absolutely right. We omitted the unrelated biomarkers in the revised introduction and instead, we referred to the metabolomics papers.

• Introduction: Line 61-62: “….no parameters have been confirmed for diagnosing and predicting hypertension.” This statement is simply not true as there are many factors previously reported on (including biomarkers) that can be used for the prediction of hypertension. Perhaps I misunderstood you. Please clarify.

Done. Thanks for your appropriate comment. You are right. We deleted this statement in the revised manuscript.

• Introduction: Line 66: What is meant with metabolomics is a practical method? It requires expensive equipment and trained staff to perform these analyses?

Done. Thanks for your precise comment. We corrected it in the revised manuscript.

• Introduction: Line 68: The reference to organic acids is quite broad as there are many organic acids that form part of multiple metabolic pathways?

Done. Thanks for your suitable comment. We revised it in the introduction section of the revised manuscript.

• Methods: Line 83: Please specify the device used to measure BP

Done. Thanks for your appropriate comment. We specified the device used to measure BP in the method section. We used Lumiscope professional aneroid sphygmomanometer with adult cuff and stethoscope to measure BP.

• Methods: For both the biochemical measurements and the metabolomics analysis the coefficient of variation should be reported.

Done. Thank you for your appropriate comment. We reported the coefficient of variation in the revised manuscript.

• Methods: What type of QCs did you incorporate in your metabolomics analyses. Was it done in batches? How did you correct for batch effects?

Done. Thanks for your comment. All materials were bought at the same time (first of the study), so batch difference did not affect the results. To ensure the reliability of results, quality control material were analyzed together with sample in each run which is briefly explained in revision.

• Methods: Line 101: Reference is made to internal standards. What was used? What concentrations?

Done. Thank you for the useful comment. Internal standards were used to compensate variation happened during sample preparation, extraction and chemical derivatization and also to decrease variability in signal intensity. The concentration for each analyte was different, briefly 3-22 µmol/L for aminoacids and 0.03-0.7 µmol/L for acylcarnitines.

• Methods: What is the level of certainty of the identification of your metabolites? Did you compare it with commercially available standards? How did you quantify the levels of your metabolites?

Done. Thank you for the comment. We used commercial standards /calibrators and prepared different dilutions to make calibration curve based on response of STD/ IS for each analyte. Using the calibration curve, the value of each analyte was quantified in each sample. To ensure the reliability of results, quality control material were analyzed together with sample in each run. To clarify the process of calibration and quality control, it is briefly explained in the revision.

• Methods: Line 121: Adjustments were made for the lipid profile? Did you include all measures as part of the lipid profile? Or only HDL? Be specific.

Done. Thanks for your excellent comment. We adjusted for age, sex, BMI, lipid profile (cholesterol, triglyceride, HDL-C), FPG, HbA1c, oral glucose-lowering drugs use, and statin use in the model 2 and we clearly stated these covariates in the method section of the revised manuscript.

• Methods: Line 121: How did you decide on FPG to adjust for instead of HbA1c?

Done. Thanks for your comment. We adjusted our model 2 for age, sex, BMI, lipid profile (cholesterol, triglyceride, HDL-C), FPG, HbA1c, oral glucose-lowering drugs use, and statin use. We did this adjustment in all of our analysis including model 2 in logistic regression analysis (S2 Table) and model 2 in our factor analysis (Fig 2). We also revised the main text, S2 Table, and Fig 2 according to the new results from model 2 adjustment.

• Results: Line 127-128: Instead of indication that each group consisted of almost half females, rather indicate that there was an equal sex distribution.

Done. Thanks for your comment. We indicated in the result section of the revised manuscript that there was an equal sex distribution instead of each group consisted of almost half females.

• Results: Line 131: I am not sure where the (p≤0.026) comes from as all the p-trend values indicated in Table 1 (with the exception of statin use) are 0.001? Did you also compare the groups in post-hoc analyses? If you did, it should be indicated in Table 1.

Done. Thank you very much for your comment. We correctly stated the p-value in the revised manuscript. We provided the results of post-hoc analyses in the Table S1. The (p≤0.026) referred to the pairwise comparison of stage 2 HTN with control and stage 1 HTN with control for the following parameters: BMI, waist circumference, FPG, HbA1C, TG, total cholesterol, and Non-HDL-C. We clearly provided all pairwise comparisons between groups in the Table S1.

• Did you adjust for statin use or the use of other medication? Perhaps that should be included as sensitivity analyses?

Done. Thanks for your appropriate comment. In the revised manuscript, we adjusted our model 2 for age, sex, BMI, lipid profile (cholesterol, triglyceride, HDL-C), FPG, HbA1c, oral glucose-lowering drugs use, and statin use. We revised the main text, figure 2 and S2 Table based on the new adjusted model 2.

• What was the rationale for inter-correlating the metabolites (Fig1). As this was not part of your original aim?

Done. Thanks for your question. As you have well mentioned, it is not an aim of this study but it is a preliminary fundamental step for further data analysis which the whole analysis is based on. The existence of enter-correlation supported the need of PCA, as it is mentioned in the method part.

• Table 2: It is not clear what exactly you are reporting. Did you compare all groups? Or did you compare all groups with the control group? I think it may be worthwhile to do the latter if you did not. Also, to just indicate that there are differences without indicating if it was higher/lower does not say much?

Done. Thanks for your promising comment. In this table, we compared all group. But now, we provided the pairwise comparisons of metabolite concentration in different groups with control group in the last columns of the table 2. In the main text, we also indicate the higher/lower level of metabolite profile in pairwise comparison of different groups with control group.

• Discussion: Line 205 & 246: I think the use of the work “upregulated” is not appropriate here. Rather say that the abundance of acylcarnitines were higher. Normally you would use the term upregulated for an enzyme and in this case if acylcarnitines are accumulated it would suggest that the beta-oxidation enzymes are not functioning optimally?

Done. Thanks for your accurate comment. We corrected this statement in the revised manuscript.

• Discussion: Line 205-207: It is indicated that beta-oxidation may not be functionally optimally. Can you speculate as to why this is the case?

Done. Thanks for your comment. It can be caused when the fatty acids uptake surpasses the rates of β-oxidation or in the cases of incomplete fatty acids β-oxidation which leads to intracellular lipids accumulation (reference 15).

• Discussion: Line 254: It is indicated that plasma is the best sample choice. It should be considered that urine can also be used and that urine is a lot less invasive to collect.

Done. Thanks for your appropriate comment, this part was deleted.

• Conclusion: Line 261: “….found the unique metabolite profile of plasma” This phrase in unclear. Also the phrase “….different stages of blood pressure” is not clear and should be clarified.

Done. Thanks for your appropriate comment. We clearly rephrased this sentence in the revised manuscript.
