# Supplementary material for: Association of plasma acylcarnitines and amino acids with hypertension: A nationwide metabolomics study
Source: PLoS One. 2023 Jan 17;18(1):e0279835. doi: 10.1371/journal.pone.0279835 (PMC9844860; doi:10.1371/journal.pone.0279835)
Supplement: Attachment — Submitted filename: PONE-D-21-34134-R1_Review.docx [file pone.0279835.s007.docx]

# PONE-D-21-34134R1

Thank you for the opportunity to review the revised manuscript by Razi et al. Thank you for addressing the comments, however there is still several issues with this manuscript.

- Despite claiming that the manuscript was revised to incorporate professional language and technical editing. There are still several language errors.
- The term “Non-HDL-C” is rather vague? What exactly is meant with Non-HDL-C?
- Based on the comments of Reviewer 1 you now added Antihypertensive drug use and in the discussion of the article you elaborate on how certain anti-hypertensive drugs can modify acylcarnitine levels. This brings about a lot of questions such as what type of drugs did your population use? Should you not consider adjusting for this in the statistical analyses? Or do a sensitivity analysis where those using antihypertensive drugs are excluded?
- Thank you for clarifying the adjustment for the lipid profile. However, adjustment for cholesterol, triglycerides, and HDL-C in one model may lead to multicollinearity as these are naturally inter-correlated. This may inflate the strength of your models. My suggestions would be to select the lipid with the most relevance to the dependent and main independent variables. This can be based on the literature or by doing bi-variate regression analysis between the dependent and main independent variables with the various lipids. Or multicollinearity should be tested and indicated in the manuscript.
- Similarly, the inclusion of both FGP and HbA1c may also lead to multicollinearity.
- Just check the following phrase in the discussion (line 275-276): “Our crude and adjusted models indicted that glycine had a protective effects against the stage 2 HTN incident.” Not only does this sentence not make sense, but the phrase “protective effects” cannot be used in the context of a cross-sectional analyses.
- Please supply the evidence for the following statement: “This study had several significant strengths including its large-scale population-based design, investigation of a large number of acylcarnitine and amino acids profile, evaluation of metabolite in plasma which is the best sample for this purpose…..”
