# Supplementary material for: Association of plasma acylcarnitines and amino acids with hypertension: A nationwide metabolomics study
Source: PLoS One. 2023 Jan 17;18(1):e0279835. doi: 10.1371/journal.pone.0279835 (PMC9844860; doi:10.1371/journal.pone.0279835)
Supplement: Attachment — Submitted filename: Response to Reviewers.docx [file pone.0279835.s008.docx]

Response to Reviewers

Reviewer #1: The authors have addressed my comments. The quality of the writing could be improved but overall I can easily understand the study including results and discussion.

Done. Thank you. We improved the quality of the writing in the revised manuscript.

Reviewer #2: Thank you for the opportunity to review the revised manuscript by Razi et al. Thank you for addressing the comments, however there is still several issues with this manuscript.

• Despite claiming that the manuscript was revised to incorporate professional language and technical editing. There are still several language errors.

Done. Thank you for your comment. The manuscript was revised to incorporate professional language and technical editing.

• The term “Non-HDL-C” is rather vague? What exactly is meant with Non-HDL-C?

Thank you for your comment. For clarity, we replaced non-HDL-C by non-HDL cholesterol in the revised manuscript and described how non-HDL cholesterol was calculated. Non-HDL cholesterol was simply calculated by subtraction of high-density lipoprotein (HDL, or "good") cholesterol number from the total cholesterol number. So non-HDL cholesterol contains all the "bad" types of cholesterol.

• Based on the comments of Reviewer 1 you now added Antihypertensive drug use and in the discussion of the article you elaborate on how certain anti-hypertensive drugs can modify acylcarnitine levels. This brings about a lot of questions such as what type of drugs did your population use? Should you not consider adjusting for this in the statistical analyses? Or do a sensitivity analysis where those using antihypertensive drugs are excluded?

Done. Thanks for your appropriate comment. You are absolutely right. We adjusted for using antihypertension drugs in the Model 2. When we included antihypertensive drugs intake in our full adjustment model, three metabolites, including C8:1, C10, and C10:1, lost their predictive value for stage 2 HTN. This indicated that the association of these metabolites with stage 2 HTN was dependent of antihypertensive drugs intake. Finally, we had three models including crude model, model 1 that adjusted for age, sex and BMI, and model 2 that adjusted for age, sex, BMI, lipid profile (total cholesterol, triglyceride, HDL cholesterol), FPG, oral glucose-lowering drugs use, statins use, and antihypertensive drugs use. We also added it to our limitations that the type of antihypertension drugs that the participants consumed was unclear.

• Thank you for clarifying the adjustment for the lipid profile. However, adjustment for cholesterol, triglycerides, and HDL-C in one model may lead to multicollinearity as these are naturally inter-correlated. This may inflate the strength of your models. My suggestions would be to select the lipid with the most relevance to the dependent and main independent variables. This can be based on the literature or by doing bi-variate regression analysis between the dependent and main independent variables with the various lipids. Or multicollinearity should be tested and indicated in the manuscript.

Done. Thank you for your appropriate comment. We tested the multicollinearity between total cholesterol, triglycerides, and HDL cholesterol and we provided the results of the multicollinearity tests in the revised manuscript. As shown, the magnitudes of correlation coefficient between total cholesterol, triglycerides, and HDL cholesterol were lower than 0.5; so, we adjusted for all lipid profile (total cholesterol, triglycerides, and HDL cholesterol) in our model due to low multicollinearity and also, because all these lipid profile (total cholesterol, triglycerides, and HDL cholesterol) independently were associated with HTN (1, 2) and metabolite concentrations (3).

• Similarly, the inclusion of both FGP and HbA1c may also lead to multicollinearity.

Done. Thank you for your appropriate comment. There was high linear relationship between FGP and HbA1c (Pearson correlation coefficient=0.805) and so there was multicollinearity between them. These variables both represent the glucose concentration. The previous evidence in the literature showed that impaired FPG only showed a 2.13-fold increased risk for HTN while Impaired HbA1C only was not found to be independently associated with HTN (4). In addition, the study by Alqudah A et al. showed that FPG had stronger correlation with various metabolites concentration than HbA1c (5). So, we only adjusted for FPG in the model 2.

• Just check the following phrase in the discussion (line 275-276): “Our crude and adjusted models indicted that glycine had a protective effects against the stage 2 HTN incident.” Not only does this sentence not make sense, but the phrase “protective effects” cannot be used in the context of a cross-sectional analyses.

Done. Thank you for your appropriate comment. You are absolutely correct. We cannot conclude a causal effect from cross-sectional study. So, we corrected this sentence in the revised manuscript, and now the revised sentence (“Our crude and adjusted models indicated that glycine was negatively associated with the stage 2 HTN.”) showed a relationship effect.

• Please supply the evidence for the following statement: “This study had several significant strengths including its large-scale population-based design, investigation of a large number of acylcarnitine and amino acids profile, evaluation of metabolite in plasma which is the best sample for this purpose…..”

Done. Thank you for your comment. We revised this statement in the revised manuscript. This study investigated the metabolomics analysis of 50 metabolites (acylcarnitines and amino acids) on 1200 participants, and to our knowledge, no metabolomics analysis study has been conducted in Iran with such a large sample size and metabolite count as this study. Besides, there have been few studies in the world with sample size of over 1200 participants that investigated the metabolomics level of over 50 acylcarnitines/amino acids.

1.	Bønaa K, Thelle D. Association between blood pressure and serum lipids in a population. The Tromsø Study. Circulation. 1991;83(4):1305-14.

2.	Tohidi M, Hatami M, Hadaegh F, Azizi F. Triglycerides and triglycerides to high-density lipoprotein cholesterol ratio are strong predictors of incident hypertension in Middle Eastern women. Journal of human hypertension. 2012;26(9):525-32.

3.	Yang P, Hu W, Fu Z, Sun L, Zhou Y, Gong Y, et al. The positive association of branched-chain amino acids and metabolic dyslipidemia in Chinese Han population. Lipids in health and disease. 2016;15(1):1-8.

4.	Geva M, Shlomai G, Berkovich A, Maor E, Leibowitz A, Tenenbaum A, et al. The association between fasting plasma glucose and glycated hemoglobin in the prediabetes range and future development of hypertension. Cardiovascular diabetology. 2019;18(1):1-9.

5.	Alqudah A, Wedyan M, Qnais E, Jawarneh H, McClements L. Plasma amino acids metabolomics' important in glucose management in type 2 diabetes. Frontiers in pharmacology. 2021;12.
